# Water matters: An assessment of opinion on water management and community engagement in the Republic of Ireland and the United Kingdom

**DOI:** 10.1371/journal.pone.0174957

**Published:** 2017-04-03

**Authors:** Alec Rolston, Eleanor Jennings, Suzanne Linnane

**Affiliations:** Centre for Freshwater and Environmental Studies, Dundalk Institute of Technology, Dundalk, County Louth, Ireland; University of Siena, ITALY

## Abstract

Internationally, water management is moving from the traditional top-down approach to more integrated initiatives focussing on community-led action. With inadequacies in previous engagement initiatives undertaken through the first cycle of River Basin Management Planning for the EU Water Framework Directive (WFD), the Republic of Ireland has only recently embraced this bottom-up approach. The attempted introduction of national charging for domestic water use in 2015 has resulted in significant public disquiet and protest movements against the national government. In April 2015 we undertook a survey of current opinion on water management and community engagement initiatives in the Republic of Ireland and the United Kingdom. A total of 520 survey responses identified that although freshwater bodies are important in peoples’ lives, respondents were typically unaware of global initiatives such as Integrated Water Resources Management and Integrated Catchment Management. Overall, 81% of respondents did not feel included in decisions about their water environment despite an overwhelming 95% believing that local communities should have a say in how the water environment is managed. However, only 35.1% of respondents stated that they would be willing to attend local water management engagement initiatives. Rather than supporting individual gain, respondents identified social gains for the local community as avenues for increasing local involvement in water initiatives. In the Republic of Ireland, a water engagement initiative that implements the national framework local delivery model should be developed and implemented. This would 1) contribute to the second round of WFD River Basin Management Planning; 2) facilitate stronger connections between local communities and their water environment; and 3) foster bottom-up initiatives that empower communities regarding local water management issues.

## Introduction

Water is essential for all life and is important for health, spiritual needs, comfort, livelihood and the world’s ecosystems [1], yet the values given to the services provided by such aquatic ecosystems can vary between individuals [2]. Climate change, population growth, intensified agricultural production and increased abstractions are some of the pressures acting on the availability of water on a changing planet [3]. Within the water sector, institutional fragmentation can result in antagonistic management actions that fail to achieve overarching goals and that often overlook the importance of maintaining healthy freshwater ecosystems [4]. An integrated approach is therefore required in water management between different sectors to achieve future action on water and sustainable development [5]: Integrated Water Resources Management (IWRM) is one approach identified to achieve such cohesion [6]. In essence, IWRM is designed to replace the traditional fragmented methods to water management with a more holistic approach that recognises the multi-faceted social, economic and environmental importance of water and society [7].

As hydrological, economic, social and environmental interdependencies occur within catchment (watershed) areas, it is within this geographical unit that integrated development and management of water resources is likely to be most successful [8]. The need to manage water from its source to sink, and the interdependence of water uses with each other and natural processes require holistic catchment-based management [9]. Integrated catchment management (ICM) is a subset of IWRM that is based on the concepts of catchments as biophysical units in which use of natural resources and ecological and water protection takes place; local community and scientific involvement is integrated; and appropriate organisational structures and policy objectives are put in place [10].

Given the size and complex nature of global water challenges, there has been a trend for moving towards a more inclusive bottom-up approach which fosters greater participatory involvement of stakeholders and builds bridges between government leaders and citizenry, driven by past failings of top-down approaches [11–15]. Whilst great progress has been made on the scientific aspects of catchment management in the Republic of Ireland, significant deficiencies in the areas of public participation and social learning need to be urgently addressed in order to increase interactions between governing agencies and the general public and to remove engagement barriers present as a result of top-down governance and enforcement of legislation [10]. As a result of the top-down approach often being seen as potentially exclusive and alienating to local people, there has been a growing acceptance of bottom-up approaches that characteristically both appreciate and incorporate local people and their local knowledge, skills, needs and experiences [13].

Community engagement, also known as public participation or civic or citizen engagement, is a planned process with the specific purpose of working with identified groups of people, whether they are connected by geographic location, special interest or affiliation, to address issues affecting their well-being [16]. Effective engagement has been shown to lead to decisions, delivery and evaluation of services that have been shaped by the relevant people and communities [17]. Many fields of work employ community engagement practices, with key goals being to build trust, enlist new resources and allies, create better communication, and improve overall outcomes as successful projects evolve into lasting and sustainable collaborations [18].

Local communities are key stakeholders in the arena of water management and the role of public participation in catchment management is recognised as an important component in delivering water-related outcomes [19, 20]. However, effectively engaging communities to produce productive outcomes is no simple task. Bottom-up, community-led catchment based approaches to land and water management provide new challenges to policy makers as community priorities vary according to local values and pressures [21]. Collaborative efforts that focus on representing and valuing diverse viewpoints, using knowledge from local groups to inform ideas and decisions, following democratic decision making processes and using dynamic forms of communication are likely to be viewed favourably by participants [22]. However, if there is no expectation of impact, participation is unlikely [23].

ICM-focussed community engagement initiatives have been a cornerstone of Australian Natural Resource Management (NRM) policy for several decades [24]. In Europe, water-related community engagement initiatives have proliferated following the implementation of the EU Water Framework Directive (WFD) (2000/60/EC). However, there is international recognition that the public participation and engagement initiatives as part of the first cycle of WFD River Basin Management Planning have been inadequate [25]. Over the past decade in the United Kingdom, community engagement has become a core component of water management as a result of the development of national policy such as the Catchment Based Approach (CaBa) [25] and the formation of the Rivers Trust and associated non-governmental organisations. In contrast, outside of the implementation of the WFD, the Republic of Ireland has lagged behind in attempting to instigate holistic, joined-up, catchment-level thinking. Irish IWRM and ICM initiatives have tended to be isolated, stand-alone projects of limited durations rather than as part of any standardised national policy initiatives.

Whilst frameworks which identify best practice methods for community engagement are freely available [26–28], only a limited number of studies have assessed the opinion of the targets of such engagement initiatives in the Republic of Ireland and the UK [22, 29–31]. The recent (April 2015) introduction of charging for consumptive water usage in the Republic of Ireland was followed by the suspension of these charges as a result of public disquiet and political pressures leading into and after the February 2016 general election. Subsequently, water management and the engagement of communities have been elevated into the national consciousness. The disparity between the Republic of Ireland and the UK in initiating joined-up, holistic catchment-level thinking that is supported by national policy identified an opportunity to also assess whether differences in opinion on water management and community engagement initiatives exist within and between the geographic localities.

In addition, assessing opinion on water management and community engagement in both the Republic of Ireland and the UK would provide a baseline in the Republic of Ireland to inform future engagement initiatives to be developed as part of the second round of WFD River Basin Management Planning. This paper outlines the results of an online survey undertaken as part of the Towards Integrated Water Management (TIMe) Project, funded by the Environmental Protection Agency (EPA) of Ireland. The project had an overarching objective to connect science, policy, managers and local communities for the integrated management of the Republic of Ireland’s water resources to assist in delivering improvements in environmental status, water quality and water management [32]. The results of this paper have provided guidance on the organisation of water-related engagement activities in the Republic of Ireland.

## Methods

A survey of 37 questions was designed and launched through the website www.surveymonkey.com to ascertain opinion on water management and community engagement in the Republic of Ireland and the constituent countries of the United Kingdom (England, Scotland, Wales and Northern Ireland) (S1 File). Prior to being launched, the survey methodology and questions were reviewed by the TIMe Project’s EPA Steering Committee to address any ethical issues. The Dundalk Institute of Technology Research Ethics Committee was also consulted and the Committee confirmed that this type of study is normally exempt from formal review following a pre-screening assessment process. Internet Protocol (IP) addresses of respondents were not collected to ensure respondent anonymity. Contact details of researchers, the reason for conducting the survey, and the uses to be made of the data were clearly stated in the introductory page of the survey. Respondents were not required to respond to every question and it was possible to exit the survey at any point. The survey questions were divided into three components: Demography; Water and the Environment; and Water Management and Community Engagement. Because of the lower number of responses from the UK compared to the Republic of Ireland (N = 105 and N = 411 respectively: note that four respondents did not identify a geographical location), and in order to compare between geographic locations where applicable, data for all of the UK regions were combined and compared against data from the Republic of Ireland.

Demographic information gathered from each respondent included gender, age range, geographic location and societal grouping (e.g. water manager; member of the public etc.). Questions on water and the environment assessed drinking water supply provision and satisfaction; frequency of visiting freshwater bodies (identified as a stream, river, lake or canal); opinion on the environmental condition of local freshwater bodies; the perception of pressures acting on the environmental condition of local freshwater bodies; and opinion on the social, environmental and economic value of freshwater bodies. Questions within the water management and community engagement section focussed on participants’ prior and current knowledge and experiences of both water management and community engagement, and assessed the level of interest in attending future water-focussed community events.

When assessing the perception of pressures acting on the environmental condition of local waterbodies, and opinion on the social, environmental and economic value of water bodies, respondents were asked to rank opinion using a differential scale e.g. Good/Bad/No Effect; or Important/Neither important nor unimportant/Unimportant. The numbers of responses for each scale were converted to proportions to account for the differing response numbers per geographic location (Republic of Ireland or United Kingdom). The differential scales were then awarded values: 3 points for Good or Important; 2 points for No Effect or Neither important nor unimportant; and 1 point for Bad or Unimportant. Each ranking value was then multiplied by the proportion of responses recorded against that value for each item. Following assessments of data normality, One-Way Analysis of Variance (with Tukey’s Post Hoc Test where appropriate) or Kruskal Wallis Tests were then undertaken on these scored values to ascertain any significant differences between items and geographic locations. Chi-square tests were used to assess statistical differences in perceived negative effects of eight pressures acting on local water bodies by comparing the proportion of respondents which identified each pressure as being a ‘bad effect’.

The survey was promoted through online media (through the TIMe Project’s Twitter and Facebook feeds) and through targeted emails. These emails were sent to community groups (such as angling and environmental groups), River Trusts, water utility companies, local authorities, and governmental and non-governmental organisations. An article promoting the survey was also featured in the Spring 2015 edition of Rural Water News, the newsletter of the Irish National Federation of Group Water Schemes which has a distribution of approximately 3,000. Group Water Schemes are community-run potable water abstraction, treatment and distribution co-operatives in Ireland which are located in areas where there is no local authority water supply system. Over 6% of the Irish population is served by either publically or privately sourced schemes [33]. Response rates to the survey distribution were not ascertained as email recipients were encouraged to pass on the survey link and information to other interested stakeholders.

It is recognised that this survey methodology had some limitations:

- Online surveys can exclude individuals with no access to a computer or have literacy problems;
- The survey distribution method likely resulted in an exclusion of certain societal demographics and is therefore not fully representative of opinion in the geographical areas surveyed; and
- Targeted email distribution likely resulted in an over-representation of individuals that already have an active interest in the water environment.

The survey was open to respondents for a total of 39 days from the period of 23^rd^ March to 30 April 2015.

## Results

### Demographics

A total of 520 responses were recorded (although not all questions were answered by each individual respondent), with a slight male bias in the sex of respondents (55.4% male: 44.6% female). However, there was disparity in geographical location in the gender balance, with a small female bias in the UK (55.6% female) and a male bias in the Republic of Ireland (58.4% male). Overall, the majority of respondents (94.6%) were over the age of 30 (31–50 years: 55.1%; 51 years or above: 39.4%), with only 5.2% and 0.2% of respondents within the 19–30 and under 18 years categories respectively. Due to the ethical implications of including data obtained from the under-18 age cohort, the data for the one respondent within this age cohort was removed from the overall dataset and excluded from further analysis. The majority of respondents (79.4%) were located in the Republic of Ireland, whilst within the UK, the greatest proportion of respondents were located in England (60.9%), followed by Northern Ireland (16.2%), Wales (15.2%) and Scotland (7.6%).

**Fig 1:**
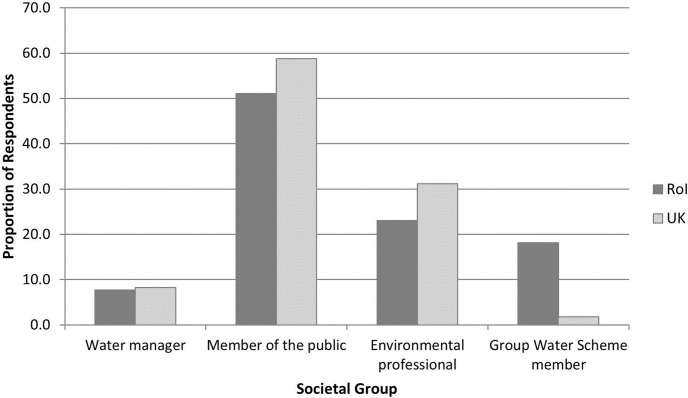
Proportion of respondents identifying themselves across societal groupings in the Republic of Ireland and the United Kingdom.

When asked to identify their societal/professional grouping, the majority of individuals in both the Republic of Ireland and the UK identified themselves as ‘Members of the public’ (51.0% and 58.7% of respondents respectively: Fig 1), followed by ‘Environmental professionals’ (23.1% and 31.2% for the Republic of Ireland and the UK respectively). A greater proportion of respondents from the Republic of Ireland identified themselves as Group Water Scheme members compared to the UK (18.1% and 1.8% respectively), largely as a result of the lack of Group Water Schemes located in the UK (one respondent from each of Northern Ireland and Wales identified themselves as Group Water Scheme Members).

### Water and the environment

In the Republic of Ireland, whilst the national water utility company, Irish Water, provided drinking water to the majority of respondents (59.1%), one-quarter of respondents (25.5%) received their drinking water from a Group Water Scheme, whilst 15.4% received their drinking water from their own private well or abstraction. This compares with 95.7% of individuals in the UK receiving their drinking water from utility companies and only 4.3% from their own private well or abstraction.

Regardless of the drinking water provider, the majority of respondents in both the Republic of Ireland and the UK were satisfied with their drinking water supply (79.7% and 84.3% respectively), although no definition of satisfaction was provided in the survey. In the Republic of Ireland, those that received their drinking water from Group Water Schemes had the highest proportion of satisfaction (93.4%), compared to supplies from private wells or private abstractions (83.3% satisfaction) and utility company provision (72.0%). In the UK, 100% of people who received their drinking water from their own private well or private abstraction were satisfied with their drinking water supply (although this sector represented only three respondents), whilst 83.7% of water utility customers expressed such satisfaction.

Overall, the primary reason why respondents were not satisfied with their drinking water supply was because the water did not taste good (Table 1). However, there was a disparity in responses based on geographical location, with the cost of water being the primary reason for dissatisfaction in the UK (representing 50% of responses), followed by the water not tasting good (28.6%). In the Republic of Ireland, the taste and look of the water were the two most popular reasons for dissatisfaction, with the cost of water only representing 8.2% of responses. Other reasons for dissatisfaction in the Republic of Ireland included the hardness of the water supplied, dissatisfaction with levels of added fluoride and chlorine, and concern regarding levels of trihalomethanes (post-disinfection treatment by-products).

**Table 1:** Reasons for respondents’ dissatisfaction with their drinking water supply.

Reason	Proportion of Respondents (%)		
	Overall	Republic of Ireland	United Kingdom
The water doesn’t taste good	41.0	43.3	28.6
The water doesn’t look good	18.0	20.6	7.1
Too expensive	16.4	8.2	50.0
Poor supply pressure	9.0	9.3	7.1
Other	(15.6)	(18.6)	(7.1)
Hardness	9.0	8.2	7.1
Addition of fluoride	3.3	4.1	0
High chlorine levels	2.5	5.2	0
High trihalomethane levels	0.8	1.0	0

**Fig 2:**
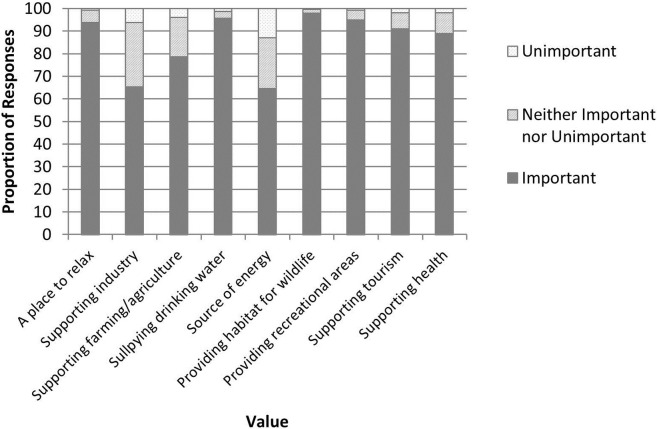
The perceived importance of values of local waterbodies.

Freshwater bodies were shown to play an important part in respondents’ lives, with 70.9% visiting a water body either daily, once or twice per week, or at least once per month (12.7%, 31.2% and 27.0% of respondents respectively). Only 3.3% of respondents stated that they never visited a freshwater body. Respondents identified that a range of aspects of the water environment were of personal importance (Fig 2), and in all cases (i.e. overall and for each geographical location), values identified as being important were significantly greater than the other two ranking options of ‘Neither important nor unimportant’ or ‘Unimportant’ (Kruskal Wallis Tests: 19.65 < H < 19.83, P < 0.001). Although fewer respondents identified local freshwater bodies as being important for supporting industry or as a source of energy, there were no significant differences of perceived importance between the different water environment values.

Overall, the majority of respondents (55.2%: 55.8% for the Republic of Ireland and 52.7% for the UK) thought that the local freshwater body that they visit was in good environmental condition (although no clarification of the meaning of ‘good’ environmental condition was made in the survey). For the 29.4% of respondents that thought their local freshwater body was not in good environmental condition, pollution caused by agriculture/farming, dumping of litter, and pollution caused by wastewater treatment plants and/or septic tanks were identified as the most common reasons for the freshwater body not being in good environmental condition (representing 19.9%, 19.1% and 15.8% of respondents respectively).

**Fig 3:**
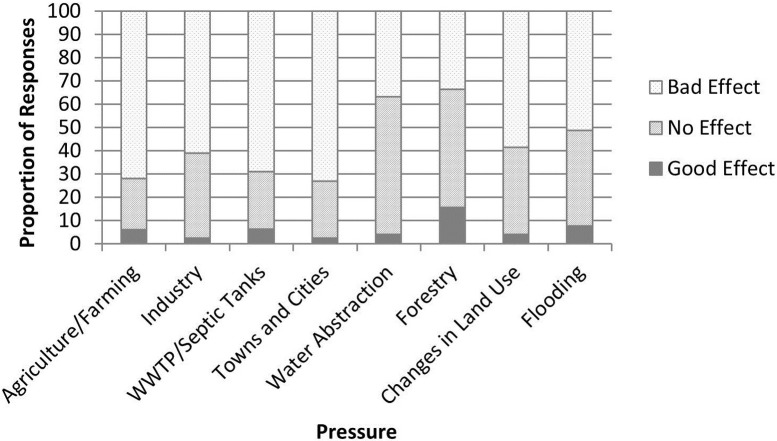
The overall perception of pressures having good, bad or no effect on local water bodies.

Overall, and for each geographic locality, a significantly greater number of respondents identified pressures acting on their water environment as having ‘bad’ effects on local freshwater bodies rather than having ‘good’ or ‘no effect’ (One-way ANOVA, for overall data F = 37.12, p < 0.001; Republic of Ireland F = 33.59, p < 0.001; and United Kingdom F = 44.03; p < 0.001: Fig 3; Table 2). Respondents identified some pressures (e.g. agriculture/farming and towns and cities) as having significantly more negative effect on waterbodies than others (Chi Squared Tests: Overall, χ^2^ = 28.9, P < 0.001; Republic of Ireland, χ^2^ = 31.9, P < 0.001; and UK, χ^2^ = 24.6, P < 0.01). Typically, water abstraction and forestry were not seen to have as large a negative impact on freshwater bodies as the other identified pressures (Fig 3).

**Table 2:** Identification of the potential effects of pressures acting on local waterbodies (ranked, scored values).

Pressure	Overall				Republic of Ireland				United Kingdom			
	Good Effect	No Effect	Bad Effect	Mean Score	Good Effect	No Effect	Bad Effect	Mean Score	Good Effect	No Effect	Bad Effect	Mean Score
Agriculture/Farming	17.3	44.6	71.9	45	17.3	41.8	73.3	44	17.2	55.2	66.7	46
Industry	6.8	73.2	60.9	47	6.7	76.3	59.6	48	7.0	62.8	66.3	45
Wastewater treatment plants/septic tanks	18.2	49.5	69.2	46	15.7	50.5	69.5	45	27.6	46.0	67.8	47
Towns and cities	6.8	49.1	73.4	43	6.8	49.8	72.8	43	7.0	46.5	74.4	43
Water abstraction	11.4	118.9	36.8	56	13.6	125.9	32.5	57	3.6	95.2	51.2	50
Forestry	46.0	102.2	33.6	61	41.2	103.1	34.7	60	63.0	98.8	29.6	64
Changes in land use	11.4	75.2	58.9	49	9.4	78.7	57.5	49	18.8	62.5	62.5	48
Flooding	22.7	82.2	51.5	52	18.5	84.2	51.7	51	38.5	74.4	50.0	54

Whilst 31.6% of overall respondents identified that everyone should be responsible for looking after the environmental condition of freshwater bodies, local authorities and state government were also identified as having such responsibility (26.4% and 17.2% of responses respectively). Although they are frequently involved in initiatives that aim to improve the environmental quality of freshwater bodies, non-governmental organisations were identified as a group that should have little such responsibility (6.2% of responses).

In order to keep freshwater bodies in good environmental condition, respondents identified the ‘polluter pays’ principle as the most popular mechanism to achieve this (30.1% of respondents), although individuals and companies that profit from the water environment, as well as ‘everybody’ were also identified to have such responsibility (20.6% and 24.5% or responses respectively).

### Water management and community engagement

**Fig 4:**
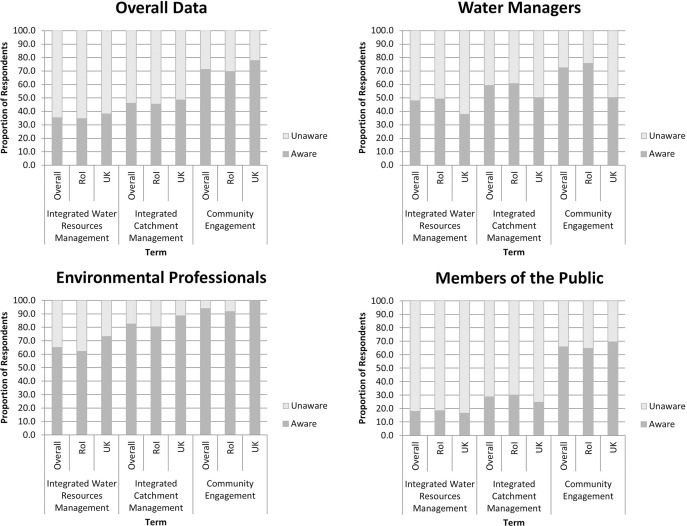
Respondent awareness of the terms ‘Integrated Water Resources Management’, ‘Integrated Catchment Management’ and ‘Community Engagement’ prior to undertaking the survey for overall data and for those who identified themselves as water managers, environmental professionals and members of the public. RoI = Republic of Ireland; UK = United Kingdom.

Respondents were asked whether, prior to completing the survey, they were aware of the terms ‘Integrated Water Resources Management’, ‘Integrated Catchment Management’ and ‘Community Engagement’. Overall, respondents were typically unaware of the terms ‘Integrated Water Resources Management’ and ‘Integrated Catchment Management’ (64.7% and 54.1% of respondents respectively), but were aware of the term ‘Community Engagement (71.1% of respondents) (Fig 4). Similar proportional responses were observed from both the Republic of Ireland and the UK. Typically, respondents who identified themselves as environmental professionals were more aware of the three terms than any other societal category: 47.5% and 59.0% of water managers were aware of the terms ‘Integrated Water Resources Management’ and ‘Integrated Catchment Management’ respectively, compared to 64.9% and 82.5% of environmental professionals respectively. The majority of both water managers and environmental professionals were aware of the term ‘Community Engagement’ (72.1% and 93.8% of respondents respectively). Members of the public were typically least aware of the terms ‘Integrated Water Resources Management’ and ‘Integrated Catchment Management’ (17.9% and 28.7% of respondents respectively). The majority of members of the public were aware of the term ‘Community Engagement’ (66.2% of respondents). Similar responses were observed between geographical locations, however, the lower response rate from the United Kingdom (e.g. n = 8 for water managers) created difficulties in drawing definitive analysis from the data.

Whilst 81.4% of respondents did not currently feel included in the decisions about their water environment (82.6% and 77.0% for the Republic of Ireland and United Kingdom respectively), nearly all respondents (95% for both geographical locations) believed that local communities should have a say in how the water environment is managed. Despite this, only 31.8% of respondents had been invited to attend a community event regarding water issues, with events being typically organised by local community groups including angling clubs, government agencies and non-governmental organisations (NGOs). NGOs were identified as being the most frequent organiser of community meetings in the UK (representing 51.4% of responses compared to only 18.2% of responses for the Republic of Ireland), reflecting the prominence of actions by organisations such as The Rivers Trusts. Such prominence was highlighted by 57.5% of UK respondents identifying local NGOs such as the Rivers Trusts as being prominent community-based groups involved with local freshwater bodies. This compares to the Republic of Ireland where Group Water Schemes were the most frequently identified community-based group (30.5% of respondents), followed by angling groups (25.6%) and other local environmental groups (23.2%). In the Republic of Ireland, more respondents identified political groups, associated with protests against the introduction of consumptive water charges, as water-active community groups than they did NGOs (8.5% and 6.1% respectively). In the Republic of Ireland, 61.4% of respondents were not aware of any community-based groups involved with local freshwater bodies or local water issues, compared to 50% of UK respondents. Overall, 34.9% of respondents were definitely interested in attending more local events on water and water management (with no difference between geographical locations), with 54.5% being non-committal on potential attendance depending on the time, location and purpose of the event.

While 68.1% of overall respondents had never volunteered to help out at any community-based water-focused event, there were geographical differences, with 71.1% of Republic of Ireland respondents and 57% of United Kingdom respondents having never undertaken such volunteer action. Despite the lack of previous volunteering experience, the majority of respondents (60.5%) stated that they would be interested in volunteering at such community-based water-focused event in the future (with no difference between the Republic of Ireland and the United Kingdom). In the Republic of Ireland, 83.7% of parents would encourage their children to become involved in community water-focused events compared to 59.7% of respondents from the United Kingdom. Lack of time (58.7% of respondents) and a lack of local initiatives to become involved in (24.5%) were cited as the most common reasons for not being involved in any such future events.

**Fig 5:**
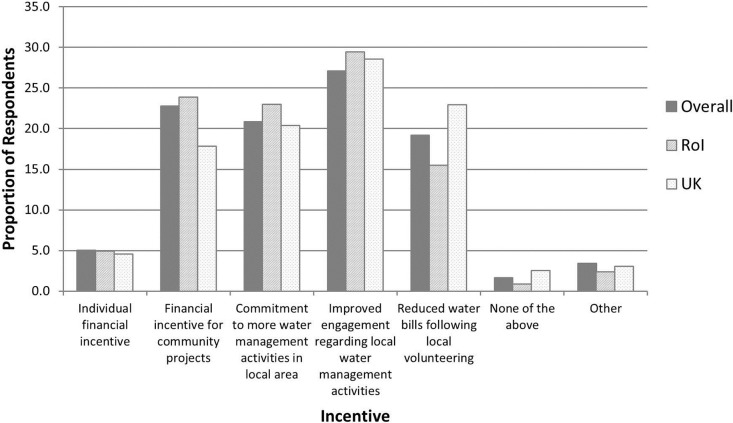
Potential incentives that may increase community involvement in water management issues. RoI = Republic of Ireland. UK = United Kingdom.

The three most common goals that were selected for increasing community involvement in water management issues were 1) improved engagement regarding local water management activities; 2) financial incentives that are invested back into local community projects; and 3) a commitment to more water management activities in the local area (Fig 5). Reduced water bills was another popular incentive, particularly in the United Kingdom, whilst other incentives suggested by respondents (through an open text box option) included greater community education/awareness, improved water quality or environmental condition, greater community ownership of projects, and improved local amenities such as fencing, walkways and signage.

Community event days and public meetings in a local venue were identified as the most effective events to supply information on water management to local communities (44.6% and 31.9% of respondents respectively). The requirement that these be locally held was highlighted by the fact that only 22.5% of respondents were willing to travel more than 20 km to attend such an event, with a travel distance of 6–10 km being the most popular distance (30.6% of respondents). The ideal frequency of such events was identified to be either 6 monthly or annually (47.0% and 34.3% of respondents respectively) in order to keep people informed on local water management issues, with little disparity between Republic of Ireland and United Kingdom respondents.

Although 61.2% of respondents believed that not enough resources were committed to improve local water management issues (with 34.0% unsure), nearly all respondents (95.3%) stated that local business should show commitment to improving the local water environment. In the Republic of Ireland, 72.3% of respondents were aware of national commitments to improve water quality under EU legislation, compared to 56.0% of respondents from the UK.

## Discussion

The global prominence of water resource management has increased over the past 20 years, and IWRM and ICM initiatives in particular have promoted public participation and community engagement. Yet few studies have investigated opinions on water management and community engagement, despite evidence for perceptions of the water environment amongst the general public (in the United Kingdom) being sparse even a decade ago [34]. Here, we undertook a survey which aimed to assess opinion on water management and community engagement in the Republic of Ireland and the United Kingdom.

The limitations in the distribution methods of this survey resulted in a lower number of responses received from the United Kingdom compared to the Republic of Ireland. This led to difficulties in directly comparing a number of results between the geographic locations and excluded comparisons between the component UK countries of England, Scotland, Wales and Northern Ireland. In addition, the limitations in the survey distribution methods meant that 1) certain socio-economic groups and individuals may have been inadvertently excluded from taking part in the survey and there is the possibility that the results presented may not be representative of these excluded communities; and 2) individuals that already held an active interest in water management and water-related community engagement activities may be over-represented. Despite these limitations, a number of key results were obtained that can be used to inform such engagement initiatives into the future. Given the disparity between the Republic of Ireland and the UK in initiating joined-up, holistic catchment-level thinking that is supported by national policy, there are surprisingly few differences in opinion between geographic localities.The likelihood of being able to engage all socio-economic groups within a community is low. Indeed, a crisis event may be necessary for some community members to engage [35], yet efforts to standardise and formalise public involvement in natural resource management initiatives can lead to community marginalisation [36]. Perceptions of what entails successful engagement can vary wildly, and even well established and internationally lauded bottom-up initiatives such as Australian Landcare have been criticised for being too ‘top-down’ in their approach [37]. In addition to facilitating wider opportunities for communities to become involved in local water management and other natural resource management initiatives, measuring and evaluating successful engagement should be a core component of a community engagement initiative, but is one that is frequently lacking [38]. Measuring the effectiveness of an engagement initiative is no easy task, and there are no standardised methods for measuring engagement success [39]. This is particularly the case with regards to assessing knowledge exchange; and the identification of indicators that focus both on the quality of participatory processes and the service delivery of an engagement process [40]. Clear definitions of the ‘community’ which is the target of engagement, and two-way communication at all stages of an engagement initiative have been shown to be critical in delivering successful engagement [41]. If successful community engagement in the water resources environment is to be implemented, both in the Republic of Ireland and in the UK, then full consideration of evaluation methodologies should be undertaken prior to the organisation of any engagement events.

The results of this survey identified the importance of local freshwater bodies to selected communities, highlighting the value that people place on waterbodies for a range of ecosystem services that they provide. These results concur with findings of other studies. For example, in the UK the majority of survey respondents (84% of 173 people) stated that the River Wear in County Durham, England, was very important in improving their quality of life, alongside other cultural services such as providing a relaxing green space, supporting recreational needs and because it provides a habitat for fish and wildlife [30]. In the Australian State of Victoria, over 90% of Victorians used waterways for beside-water recreational use such as hiking, walking, picnics and barbecues, with frequency of such recreational activities being directly linked to high aspirations of local waterway health [42]. Because these services sometimes compete against each other, the management of water is becoming an increasingly political process [43].

A collaborative approach to catchment (watershed) management has been identified as being an important learning process for both communities and governing agencies, with the need to better understand the process of catchment management being the primary reason for stakeholder involvement in an Integrated Catchment Management initiative (the Lower River Wear Pilot Project, England) [22]. Other important reasons cited included: to increase initiatives being undertaken in the locality; to increase personal networks; and to actively participate in a local initiative. In the results presented in the current paper, respondents’ reasons for becoming involved in local water-related projects focussed on social gains for the local community (such as increased funding and engagement for local initiatives) rather than for individual personal gain. Members of the public have previously been shown to be willing to pay to fund biodiversity conservation, depending upon whether the biodiversity outcomes were visible and local and that any achievements were well publicised [44]. Focussing the importance of engagement at a local level where relevance and visibility of the impact of involvement has been identified as a key issue for stakeholders and members of the public regarding implementation of the EU WFD [45]. However, the level of stakeholder engagement under the WFD in Ireland has been criticised [46], and there is a general recognition internationally that the second round of River Basin Management Planning should be much more inclusive of community partnerships and stakeholder engagement [25]. Yet, this survey identified that only 35.1% of respondents were willing to attend such water-related local engagement events. Such a low willingness to attend such events highlights the difficulties in obtaining representative community opinion despite a willingness from agencies to engage on key water management matters. If only 35% of respondents who already have an interest in water matters are willing to attend engagement events, attendance rates from other societal demographics are also likely to be low.

The majority of respondents in this study clearly perceived that most pressures result in ‘bad effects’ on their local freshwater bodies, reflecting the primary areas of concern that people have for those local freshwater bodies that are not considered to be in good environmental condition. Pollution from agriculture/farming, wastewater treatment plants and septic tanks, along with littering and poor water quality were the key issues selected as afflicting local water bodies. This supports the finding that the dominant water-related concern in both the Republic of Ireland and the UK was that of water quality (for consumption, commercial and recreational activities) [29,34]. Pollution, waste disposal, nutrient enrichment/eutrophication and agriculture were important public concerns in the Republic of Ireland [29]. These concerns seem well-founded as agriculture and municipal point sources are the primary causes of pollution at polluted river sites across the Republic of Ireland [47]. Water abstraction and forestry were both identified by survey respondents to have lesser impacts on local water bodies than other pressures. Larger water abstractions in the Republic of Ireland and the UK are typically licensed (if above 10 m^3^ and 20 m^3^ per day respectively), however accumulative unlicensed abstractions and over-allocation of abstraction licences can result in reduced surface water flows and reduced groundwater levels which can in turn impact on the environmental quality of the water resource, local freshwater bodies and groundwater dependent ecosystems [48–51]. If not suitably managed, commercial coniferous forestry has the potential to significantly impact aquatic environments through sedimentation from clear felling, planting, harvesting and road construction; and eutrophication through fertilisation of planted areas [52]. However, those respondents that identified forestry as having a good impact on local water bodies are also correct, as riparian forest buffer zones where implemented have been shown to remove sediment and provide erosion control, protect water quality, moderate shade and water temperature, maintain habitat structural diversity and improve landscape quality [53]. Differing individual perspectives can influence responses. For example, a wastewater treatment plant may be simultaneously perceived to be having both good and bad effects on a local water body. From one perspective, the discharge from a wastewater treatment plant can be perceived as having a bad effect on the receiving water body. Alternatively, the treatment of waste may be seen as being beneficial to the same water body because of the higher nutrient and chemical levels that would be present in the water body if no wastewater treatment process was in place.

Whilst Integrated Water Resources Management and Integrated Catchment Management have both been promoted globally over the past 20 years as processes that are inclusive, holistic and encourage community involvement of water management [7, 15, 54, 55–58], the majority of survey respondents were unaware of IWRM and ICM as water management initiatives. This is a significant concern, given the targeted distribution of the survey to societal groups that were already likely to have a vested interest in water resources management. Despite the increasing volume of engagement activities and the progress towards bottom-up driven catchment science, there is little evidence that the widespread advocacy and adoption of community engagement and partnership approaches have involved substantial real power sharing [12]. This may be due to governments having a tendency to retain control of funding, service contracts and regulation, and that the capacity and motivation of citizens to participate effectively, or create alternative forums, is a weakness in community engagement strategies [12]. The fact that 81% of the study survey respondents do not feel included in decisions regarding their water environment supports the conclusion that participation strategies in both the Republic of Ireland and the United Kingdom are yet to fully engage communities beyond the occasional one-off initiative or project. This is despite an overwhelming 95% of respondents stating that communities should be involved in decisions regarding the water environment. However, the disparity remains between respondents stating that communities should be involved in local water management decisions, and yet only 35.1% of respondents stating that they would be willing to attend local water management engagement events.

As both IWRM and ICM often require their initial implementation to be a top-down driven process, it is somewhat surprising that approximately half of respondents who identified themselves as water managers in both the Republic of Ireland and the United Kingdom were unaware of these terms. Given the lower number of survey responses from the UK (and subsequently the small number of respondents who identified themselves as water managers), strong conclusions cannot be drawn from the UK data in this instance. However, this result does highlight the need for increased resources and publicity for IWRM and ICM principles in order to raise awareness and increase the potential success of engagement initiatives, particularly in the Republic of Ireland, and for further assessments of IWRM and ICM awareness in the UK. Although the majority of survey respondents were aware of the term ‘community engagement’, members of the public (to which such engagement is directed) were typically unaware of IWRM and ICM, identifying a paradox for these types of initiatives in the Republic of Ireland and the UK. In Australia, where IWRM and ICM initiatives have been implemented since the 1980s [24], 36% of Victorians stated that they regularly took part in local projects to help protect waterways [42]. This compares favourably to the 43% of UK respondents in this present study who had previously volunteered in community-based water-focussed events, whilst in the Republic of Ireland, only 29% of respondents were involved in such events, although the frequency of such involvement in the UK and the Republic of Ireland was not identified.

The timing of the release of this survey (April 2015) coincided with the initial implementation of charging for water use in the Republic of Ireland following the recent establishment of the new state water utility company, Irish Water (note: the national proportion of the Irish population who receive their potable water from Irish Water is 82.1% [59] and, therefore, this survey is an under-representation of the utility company’s customer base). Significant public and political opposition to the formation of Irish Water and the formal charging of water use has resulted in water management and water resources being propelled into the national consciousness at a level never before experienced in the Republic of Ireland. Yet of the 21% of people in the Republic of Ireland that were dissatisfied with their water supply, only 8.2% cited the cost of water as a reason for their dissatisfaction. This compares to the UK where of the 18.5% of respondents that were not satisfied with their water supply, 50% cited cost as a reason for their dissatisfaction. This may be a result of the period of time that water charges have been in place in the UK, or the level of pricing in the UK. The success of national-level protests against water charges in the Republic of Ireland, which resulted in a suspension of water charges following the formation of a minority government after national elections in February 2016, was due to a genuine grassroots and local movement that united people’s voices at a national level to become too visible to be ignored by the State government [60]. Subsequently, price may feature as a key component of consumer water supply dissatisfaction in the Republic of Ireland into the future, once a government-appointed review of water charges is completed in 2017.

## Conclusions

There are seemingly few differences in opinion between survey respondents from the Republic of Ireland and the United Kingdom, despite the different timeframes of implementation of IWRM and ICM principles within the geographic localities. Although there was a much smaller response rate from the UK compared to the Republic of Ireland (perhaps reflecting the high level of interest in water currently in the Republic of Ireland as well as the method of promotion of the survey being more targeted towards Irish audiences), the survey results do allow a certain comparison of current opinion between the geographic localities.

The emphasis on the local aspect of water management and community engagement was highlighted by the majority of respondents being willing to travel less than 20 km to attend an event on water management. This restriction would present difficulties for organisations and agencies looking to develop community involvement in water management as available resources will limit the number of possible activities and may therefore restrict the volume of involvement at the local level. Careful planning of engagement activities will therefore be necessary, perhaps with a focus on regional areas that require priority water management activities as an initial step in developing community involvement in such actions at the national level.

The results of this survey identified opinion on water management and community engagement during a period of significant interest in water issues in the Republic of Ireland. The political nuances regarding water management that are likely to develop in the near future in the Republic of Ireland [60] mean that the opportunity to keep water and water resource management high in the public consciousness in the coming years remains strong. In the Republic of Ireland, this opportunity should be used to develop and implement a water engagement initiative that implements the national framework, local delivery model. Given appropriate resourcing, these initiatives would strongly contribute to the second round of WFD River Basin Management Planning, facilitating stronger connections between local communities and their water environment and fostering bottom-up initiatives that empower and give ownership of local water management issues to these communities. In the UK, further assessment of local involvement in water resources management is required, particularly in assessing the socio-economic benefits of the catchment-based approach. This approach is supported by national policy, with on-ground delivery facilitated by both government agencies and NGOs such as the Rivers Trust. The UK Rivers Trust model is currently being examined for implementation in the Republic of Ireland in order to increase bottom-up delivery of water resources management. However, this survey has identified that significant challenges remain with regards to the involvement of communities in local water resources management and awareness-raising of international processes such as IWRM and ICM in both the Republic of Ireland and the UK.

## Supporting information

S1 File Survey questions. (PDF)
