# Supplementary material for: Water matters: An assessment of opinion on water management and community engagement in the Republic of Ireland and the United Kingdom
Source: PLoS One. 2017 Apr 3;12(4):e0174957. doi: 10.1371/journal.pone.0174957 (PMC5378399; doi:10.1371/journal.pone.0174957)
Supplement: S1 File — (PDF) [file pone.0174957.s001.pdf]

1. Welcome to this survey on Water and Community Engagement

The Towards Integrated Water Management (TIMe) Project is funded by the Environmental Protection Agency of Ireland and managed through Dundalk Institute of Technology, Ireland. The project aims to connect science, policy, managers and local communities for the integrated management of Ireland's water resources to help deliver improvements in environmental status, water quality and water management.

As part of the project we are undertaking a survey of opinion on current water resource management and community engagement throughout the Republic of Ireland, the United Kingdom and Northern Ireland, with the aim of identifying a focus for future community engagement processes.

There are 38 questions and the survey will take less than 15 minutes to complete.

The information you supply will be anonymous and you will not be identified in any report or article that is published as a result of this survey.

Thank you for your time and for helping us work towards improving the management of water and engaging communities.

Further information on the TIMe Project can be found at the following locations:

Web: https://www.dkit.ie/cfes/research-projects/lake-catchment-management/towards-integrated-water-management-time-project

Twitter: TIMe_Project@Water_DundalkIT

Facebook: www.facebook.com/TowardsIntegratedWaterManagementProject

Postal Address:
Towards Integrated Water Management Project,
Centre for Freshwater and Environmental Studies,
Dundalk Institute of Technology,
County Louth,
Ireland

2. Demographics

1. What is your gender?

- Male
- Female

2. What is your age range?

- Under 18
- 19-30
- 31-50
- 51 or above

3. Which of the following best describes you?

- Water Manager
- Member of the Public
- Environmental Professional
- Group Water Scheme Member

4. Where do you live?

- Republic of Ireland
- Northern Ireland
- England
- Scotland
- Wales

5. If you live in the Republic of Ireland, please select your province

- Leinster
- Munster
- Connaght
- Ulster

3. Water and the Environment

6. Who provides your drinking water supply?

- ◯ Water utilities provider (e.g. Irish Water, Northumbrian Water, etc.)
- ◯ Group Water Scheme
- ◯ Own private well or abstraction

7. Are you satisfied with your drinking water supply?

- ◯ Yes
- ◯ No

8. If you are not satisfied with your drinking water supply, why is this (you may select more than one option)?

- ☐ Too expensive
- ☐ Poor supply pressure
- ☐ The water doesn't taste good
- ☐ The water doesn't look good

Other (please specify)

9. How often would you visit a waterbody (any stream, river or lake)?

- ◯ Daily
- ◯ Once or twice a week
- ◯ At least once a month
- ◯ Every 2-3 months
- ◯ Once or twice a year
- ◯ Never

10. Do you think that the waterbody (stream/river/lake) that you visit most often is in good environmental condition?

- ◯ Yes
- ◯ No
- ◯ I don't know

11. If you answered no to the previous question, why do you think the waterbody is not in good environmental condition (you may select more than one option)?

- [ ] I think the water is of poor quality
- [ ] There are few plants and animals
- [ ] The water smells bad
- [ ] I think industry is polluting it
- [ ] I think agriculture/farming is polluting it
- [ ] I think wastewater treatment plants and/or septic tanks are polluting it
- [ ] There are no fish to catch
- [ ] People throw lots of rubbish into it
- [ ] I think too much water is abstracted from it

Other (please specify)

12. Who do you think is responsible for looking after the environmental condition of streams, rivers and lakes (you may select more than one option)?

- [ ] State Government
- [ ] Local Authority
- [ ] Group Water Scheme
- [ ] The General Public
- [ ] Non-Governmental Organisations
- [ ] Everybody

13. Who do you think should pay to keep streams, rivers and lakes in good environmental condition (you may select more than one option)?

- [ ] The tax payer
- [ ] Those that pollute the waters
- [ ] The Government
- [ ] Those that profit from the water environment
- [ ] Everybody

Other (please specify)

14. Do you think the following have good, bad or no effect on the waterbody that you most frequently visit?

	Good	Bad	No effect
Agriculture/Farming	◯	◯	◯
Industry	◯	◯	◯
Wastewater treatment plants and/or septic tanks	◯	◯	◯
Towns and Cities	◯	◯	◯
Water abstraction	◯	◯	◯
Forestry	◯	◯	◯
Changes in land use	◯	◯	◯
Flooding	◯	◯	◯

15. How important are the following aspects of the water environment to you?

	Important	Neither important nor unimportant	Not important
They are a nice place to spend time and relax	◯	◯	◯
They support industry	◯	◯	◯
They support farming/agriculture	◯	◯	◯
They supply drinking water	◯	◯	◯
They are used as an energy source	◯	◯	◯
They provide places for wildlife, for example fish and birds	◯	◯	◯
They provide areas for recreation	◯	◯	◯
They support tourism	◯	◯	◯
They support health	◯	◯	◯

16. Are there any other reasons why you value your water environment?

17. Are there any changes you would like to see made to your local waterbodies to improve them for people and wildlife?

4. Water Management and Community Engagement

18. Prior to starting this survey, had you heard of the term Integrated Water Resource Management?

- ◯ Yes
- ◯ No

19. Prior to starting this survey, had you heard of the term Integrated Catchment Management?

- ◯ Yes
- ◯ No

20. Prior to starting this survey, had you heard of the term Community Engagement?

- ◯ Yes
- ◯ No

21. Do you feel included in the decisions about your water environment?

- ◯ Yes
- ◯ No

22. Should local communities have a say in how the water environment is managed?

- ◯ Yes
- ◯ No

23. Have you ever been invited to attend a community event regarding water issues?

- ◯ Yes
- ◯ No

24. If you have previously been invited to a community event regarding water issues, who organised the event?

- ◯ Government Agency
- ◯ Community Group
- ◯ Non-Governmental Organisation
- ◯ I don't know

Other (please specify)

25. Are you aware of any community-based groups which are involved with local waterbodies or water issues?

- ◯ No
- ◯ Yes

Please provide the name of the group(s)

26. Would you be interested in attending more events on water and water management in your area?

- ◯ Yes
- ◯ No
- ◯ Maybe (depending on time, location and purpose)

27. How far would you be willing to travel in order to attend an event on water and water management in your area?

- ◯ Less than 5 km
- ◯ 6-10 km
- ◯ 11-20 km
- ◯ 21-50 km
- ◯ More than 51 km

28. Have you ever volunteered to help out with any community-based water-focussed events?

- ◯ Yes
- ◯ No

29. Would you be interested in volunteering to help with any future community-based water-focussed events?

- ◯ Yes
- ◯ No

30. What may stop you being involved with any future community-based water-focussed events?

- ◯ No time
- ◯ Not interested
- ◯ Lack of previous outcomes from volunteering
- ◯ Lack of local activities to become involved in

31. If you have children, would you encourage them to become involved in community water-focussed events?

- ◯ Yes
- ◯ No
- ◯ Maybe

32. What incentives do you think might increase community involvement in water management issues (you may select more than one option)?

- ☐ Individual financial incentive
- ☐ Financial incentive that is put back into local community projects
- ☐ Commitment of increased water management activities in your local area
- ☐ Improved engagement regarding local water management activities
- ☐ Reduced water bills following involvement in local voluntary water management activities
- ☐ None of the above

Other (please specify)

33. What type of event do you think supplies the best information on water management to local communities?

- ◯ Public meeting in a local venue
- ◯ One-on-one discussions with relevant people
- ◯ Community event days
- ◯ Conference/workshop

Other (please specify)

34. How frequently should community events occur to keep people informed on water management issues?

- ◯ Weekly
- ◯ Monthly
- ◯ Six monthly
- ◯ Annually
- ◯ Every 2-3 years

35. Are you aware of national commitments to improve water quality under EU legislation?

- ◯ Yes
- ◯ No

36. Are enough resources committed to improve local water management issues?

- ◯ Yes
- ◯ No
- ◯ I don't know

37. Should local businesses show commitment to improving the local water environment?

- ◯ Yes
- ◯ No

38. Do you have any further comments regarding water management and community engagement?
